# *Staphylococcus aureus* biofilm organization modulated by YycFG two-component regulatory pathway

**DOI:** 10.1186/s13018-018-1055-z

**Published:** 2019-01-08

**Authors:** Shizhou Wu, Fuguo Huang, Hui Zhang, Lei Lei

**Affiliations:** 1 Department of Orthopedics, West China Hospital , Sichuan University, No. 37 Guoxue Alley, Wuhou District, Chengdu City, 610041 Sichuan People’s Republic of China; 2 State Key Laboratory of Oral Diseases, National Clinical Research Center for Oral Disease, Department of Preventive Dentistry, West China Hospital of Stomatology, Sichuan University, No. 14 Renmin South Road, Wuhou District, Chengdu City, 610041 Sichuan People’s Republic of China; 3 West China Hospital, West China Medical School, Sichuan University, Chengdu, People’s Republic of China

**Keywords:** Osteomyelitis, Staphylococcus aureus, Two-component regulatory system, MRSA, Biofilm

## Abstract

**Background:**

*Staphylococcus aureus* (*S. aureus*) infection accounts for more than 50% of the osteomyelitis cases. Currently, methicillin-resistant *S. aureus* (MRSA) strains present an urgent medical problem. The YycFG two-component regulatory system (TCS) can allow bacteria to rapidly adapt to physical, chemical, and biological stresses. To define the role of YycFG in modulation virulence of *S. aureus* in osteomyelitis, we isolated clinical MRSA strains and compared these with ATCC29213 methicillin-sensitive *S. aureus* (MSSA).

**Methods:**

In the present study, 13 MRSA strains from chronic osteomyelitis tissues were isolated. The in-depth sequencing of 16S rRNA amplicons of the samples was conducted. Bacterial growth was monitored, and biofilm biomass was determined by crystal violet microtiter assay. Furthermore, quantitative RT-PCR analysis was adopted to identify the expression of *yycF/G/H* and *icaA/D* in MRSA and MSSA strains. Analysis of variance with one-way ANOVA was used for statistical analysis.

**Results:**

The in-depth sequencing of 16S rRNA amplicons of the clinical samples indicated a polymicrobial infection, with the phylum Firmicutes made up 13% of the microbial population. The MRSA strains showed an accelerated growth rate compared to the MSSA strains. Of note, MRSA biofilms showed an accumulation of an intercellular polysaccharides matrix and enhanced biomass upon microscopic examination. Furthermore, MRSA strains had a higher expression of the *yycF/G/H* and *icaA/D* genes and adhesion force.

**Conclusions:**

These data suggested the roles of intercellular polysaccharide in *S. aureus* pathogenesis, indicating a possible association between YycFG pathways and MRSA strain virulence.

**Electronic supplementary material:**

The online version of this article (10.1186/s13018-018-1055-z) contains supplementary material, which is available to authorized users.

## Introduction

Osteomyelitis is a progressive infection of the bone, resulting in destruction of bone tissue and bone necrosis, eventually developing into a chronic or persistent condition [1]. Chronic osteomyelitis is the prominent type of osteomyelitis with high recurrent rates, which may result from tenacious biofilms formed by *Staphylococcus aureus* (*S. aureus*), *Streptococcus pyogenes*, *Streptococcus pneumonia*, mycobacteria, and even fungi [2]. Particularly, *S. aureus* is the most commonly isolated pathogen in any type of osteomyelitis [3]. The low metabolic rates, adaptive stress responses, and decelerated rates of cell division of the deeply embedded bacteria, factors which contribute to resistance against antimicrobial agents, can be attributed to biofilms that act as diffusion barriers against those agents [4].

*S. aureus*, one of the most ubiquitous microorganisms in nature, accounts for more than 50% of the osteomyelitis cases [5]. The annual incidence of invasive bone and joint methicillin-resistant *Staphylococcus aureus* (MRSA) infections accounts for 2.8 to 43% of all invasive MRSA infections which make up 1.6 to 29.7 cases per 100,000 osteomyelitis cases [6]. In addition to the *mecA* gene which induces resistance to almost all β-lactam antibiotics, it is the capacity of *S. aureus* to form multilayered biofilms that enables it to thrive in the host, often leading to chronic conditions [7].

The YycFG two-component regulatory system (TCS), specific to low G+C Gram-positive bacteria such as *Bacillus subtilis*, *S. aureus*, *Enterococcus faecalis*, and *Streptococcus mutans*, mediates the synthesis of biofilms that allows bacteria to rapidly adapt to physical, chemical, and biological stresses. YycG is a sensor protein with histidine kinase activity, and YycF is a cognate response regulator that cooperates with the former. Mutations in the YycFG TCS have been associated with resistant and persistent infections [2, 8]. In *B. subtilis*, the genes *yycFG* originate from a part of large operon that comprises *yycFGHIJ* [9]. Besides, the *yycH* and *yycI* genes have been revealed to interact with *yycG* expression [10]. Biofilm organization is associated with the genes of *ica* locus including the *icaABCD* genes which encode the vital protein polysaccharide intercellular adhesion (PIA) [11]. In particular, *icaA* as an operon for enzyme in PIA synthesis encoding *N*-acetylglucosaminyltransferase and *icaD* play an essential role for biofilm synthesis [12, 13]. In this study, we tracked the MRSA from chronic osteomyelitis cases and methicillin-sensitive *Staphylococcus aureus* (MSSA) strains to investigate the potential roles of YycFG TCS components.

## Methods

### Ethics statement and clinical specimens

The study was approved by their Ethical Committee. The informed consent forms were signed prior to the study by the participants, all of whom were over 18 years old. Thirteen patients (5 females and 8 males; mean age 41.5 years; range 23–62 years) who were defined as chronic osteomyelitis were enrolled in this investigation (Additional file 1: Table S2). MRSA clinical isolates were obtained and identified from all these cases. Samples were resected from diseased tissues like dead bone and medullary pus according to a validated protocol designed to minimize cross contamination during the surgery [14]. Instrumental contact with the skin was avoided, and no specimens were taken from cutaneous ulcers or sinuses [15]. Part of the samples was used for microbial culture and histological identification, and the remaining were promptly stored in 25% glycerol at − 80 °C for further analysis. The antimicrobial susceptibility of those clinical MRSA isolates was identified using antibiotic twofold method at the Department of Laboratory Medicine, West China Hospital [16].

### DNA extraction, sequencing, and bioinformatics analyses

Total DNA was extracted and purified. An amplicon library from clinical specimens was created by PCR amplification with unique barcoded primers specific to the 16S rRNA V3-V4 gene region, the 338F and 806R primer pair [17] (Additional file 1).

For bioinformatics analysis, quality control, error correction, and chimera removal were first performed. Briefly, the sequence reads with unknown bases (“N”) were discarded, and high-quality reads were selected for analysis. The sequences were first clustered into operational taxonomic units (OTUs), assigned as per the RDP classifier (trained by a customized version of the comprehensive Silva database) [18], using UCLUST with a 97% identity threshold [19]. An OTU network was generated and imported to UCLUST [20], and the relative abundance of each taxon in the chronic osteomyelitis specimens was calculated.

### Bacterial strains and growth conditions

The bacterial strains and primers used in this study are listed in Additional file 1: Table S1**.** The MSSA ATCC29213 strain which was one of common sensitive reference strains [21] was provided by the Department of Laboratory Medicine (West China Hospital, Sichuan University, Chengdu, China) while the clinical MRSA strains were isolated from clinical specimens described above. Pure growth of single clones was achieved on conventional Baird-Parker (BP) agar [22], and the colonies were sequenced for strain identification (Additional file 1). *S. aureus* strains were cultured [23] (Additional file 1).

### Isolation of RNA and cDNA reverse transcription for RT-PCR assays

Total RNA was extracted from cells harvested at mid-exponential phase [23] (Additional file 1). Any remaining DNA contamination was assessed by PCR amplification using 16sR gene primers (Additional file 1: Table S1) and agarose gel electrophoresis. The purity (A260/A280) and concentration of RNA were determined using a NanoDrop 2000 Spectrophotometer (Thermo Scientific). The purified RNA was reverse transcribed to cDNA with random hexamer primers or gene-specific primers (Additional file 1: Table S1) using the RevertAid First Strand cDNA Synthesis Kit (Thermo Scientific).

### Transcription analysis by quantitative RT-PCR

All primers used for RT-PCR were obtained commercially (Sangon Biotech, Shanghai, China) and are shown in Additional file 1: Table S1. The conditions for real-time PCR are described in Additional file 1. Threshold cycle values (CT) were quantified, and the expression of each gene was normalized relative to that of 16sR gene used as an internal reference. Data were calculated according to the 2^−ΔΔCT^ method [24].

### Detection of bacterial growth and analysis of biofilm structure

*S. aureus* biofilm growth was established [23]. For scanning electron microscopy (SEM), the biofilm samples were prepared and observed with a scanning electron microscope (Inspect Hillsboro, Additional file 1). For biomass and structural assessment, the biofilms were labeled with SYTO 9 (Invitrogen, Carlsbad, CA, USA) and observed under a confocal laser scanning microscope (CLSM, FV1000; Olympus Corporation, Tokyo, Japan) at × 40 magnification. The three-dimensional reconstruction of the imaged biofilms was analyzed, and their biomass was quantified using Imaris 7.0 software (Bitplane, Zurich, Switzerland). The procedure was repeated three times for five randomly selected fields of each specimen. Bacterial adhesion force in the biofilms was assessed by atomic force microscopy (AFM). Briefly, the *S. aureus* biofilms were rinsed twice with PBS buffer and dried in air at room temperature, and the AFM procedures were performed using an SPM-9500J2 (Shimadzu, Tokyo, Japan) in the contact mode. The bacterial adhesion forces were calculated [25], and for each measurement, the probe was positioned over the biofilm surface and ten force cycles were recorded for five randomly selected bacterial cells [26].

### Crystal violet microtiter assay for determining biofilm biomass

The biomass of *S. aureus* biofilms was determined by crystal violet (CV) assay as previously described [27]. Biofilms obtained after 24 h were air dried and stained with 0.1% (*w*/*v*) crystal violet for 15 min at room temperature. The bound dye from the stained biofilm cells was solubilized with 1-μl destaining solution (ethanol/acetone = 8:2). The destaining solution was then transferred into a new plate, and biofilm formation was quantified by measuring the OD of the solution at 550 nm. The biomass of *S. aureus* biofilms included three biological replicates, and the procedure was technically replicated in three times.

### Data analysis

Statistical analyses were performed using SPSS 16.0 (SPSS Inc., Chicago, IL, USA). The Shapiro-Wilk test was used to determine whether the data were normally distributed, and Bartlett’s test was used to assess the homogeneity of variances. For parametric testing, one-way ANOVA was used to detect the significant effects of variables followed by the Student-Newman-Keuls test to compare the means of each group. The differences of the means of data were considered significant if the *p* values were < 0.05.

## Results

### Phylogenetic distribution of bacteria and species diversity

**Fig. 1:**
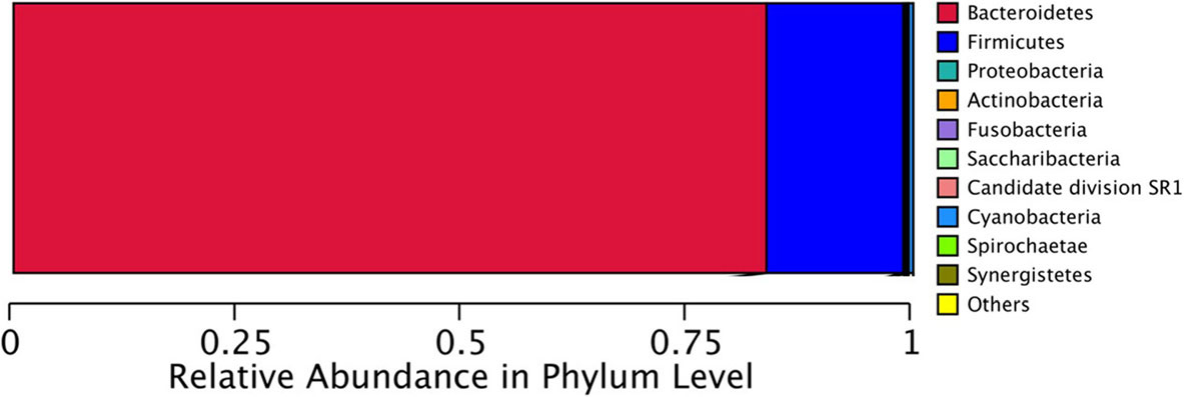
Relative abundance of phyla in the chronic osteomyelitis specimens. Taxtree for percentages of all samples. Differential microbiota compositions. The size of the column represents the relative abundance of the classification. Total DNA was extracted from clinical specimens. An amplicon library was created by PCR amplification with unique barcoded primers specific to the 16S rRNA V3-V4 gene region which contained the 30-mer 5′-end adapter sequence required for Illumina Hiseq System sequencing

After obtaining high-quality sequences, the number of reads for further analysis of each sample ranged from 1861 to 11,810 with an average length of 450 bp. The sequencing of 16S rRNA amplicons revealed several bacterial species, encompassing five phyla and eight genera, indicating a polymicrobial infection in chronic osteomyelitis specimens. The phylum *Bacteroidia* constituted the major part (around 82%) of the pathogenic flora while the phylum *Firmicutes* made up 13% (Fig. 1). Taken together, the chronic osteomyelitis-associated microbiota profile indicated a polymicrobial infection that was not restricted to the *Staphylococcus aureus* belonging to the phylum *Firmicutes*.

### Altered morphology of MRSA on bacterial growth and biofilm formation

**Fig. 2:**
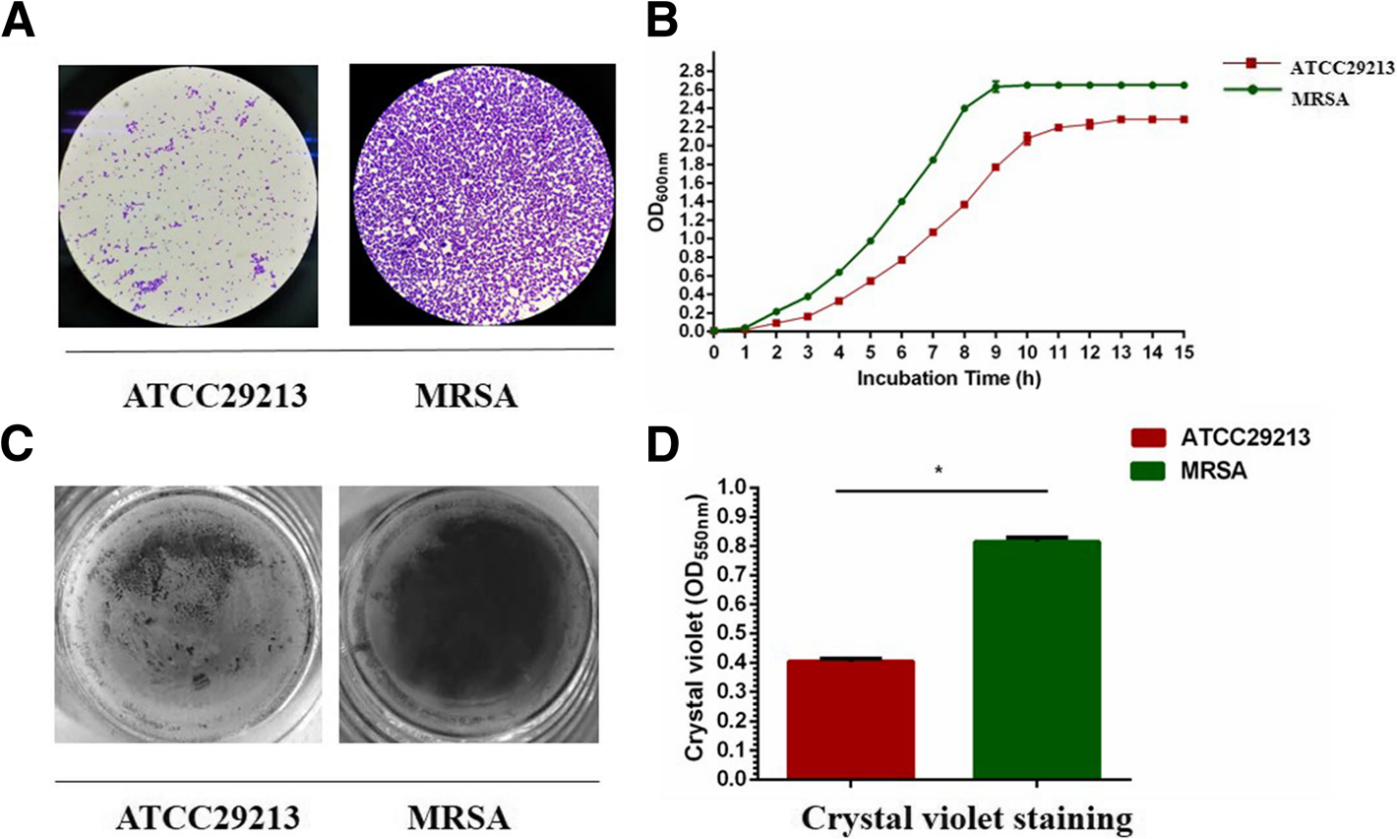
Initial comparison of MSSA and MRSA on the growth and morphology of *S. aureus*. **a** Gram’s stain for initial MRSA identification. **b** Comparison of MSSA and MRSA on bacterial growth. *S. aureus* ATCC29213 and MARSA were grown in TSB medium at 37 °C. **c** *S. aureus* 24-h biofilms grown in TSB broth containing 0.5% glucose. Biomass was quantified by crystal violet staining and the MRSA strains form more robust biofilm. **d** For crystal violet microtiter assay for determining biofilm biomass. Data represent ten biological replicates and are presented as the mean ± standard deviation (*p < 0.05, *n* = 10)

Clinical MRSA strains were identified by Gram’s staining (Fig. 2a) and 16S rRNA sequencing (Additional file 1). The growth curves of MSSA and MRSA strains were compared in three independent experiments. The entry into log phase was significantly delayed by 4 h in the MSSA ATCC29213 strain compared to the MRSA strains (Fig. 2b). MSSA biofilm formation was also decreased by 50% compared to MRSA (Fig. 2c, d). The altered phenotype of the MSSA strain is consistent with their easily disrupted biofilms and decreased EPS matrix accumulation.

### MRSA strains show elevated expression of virulence-associated genes and EPS matrix accumulation in biofilms

**Fig. 3:**
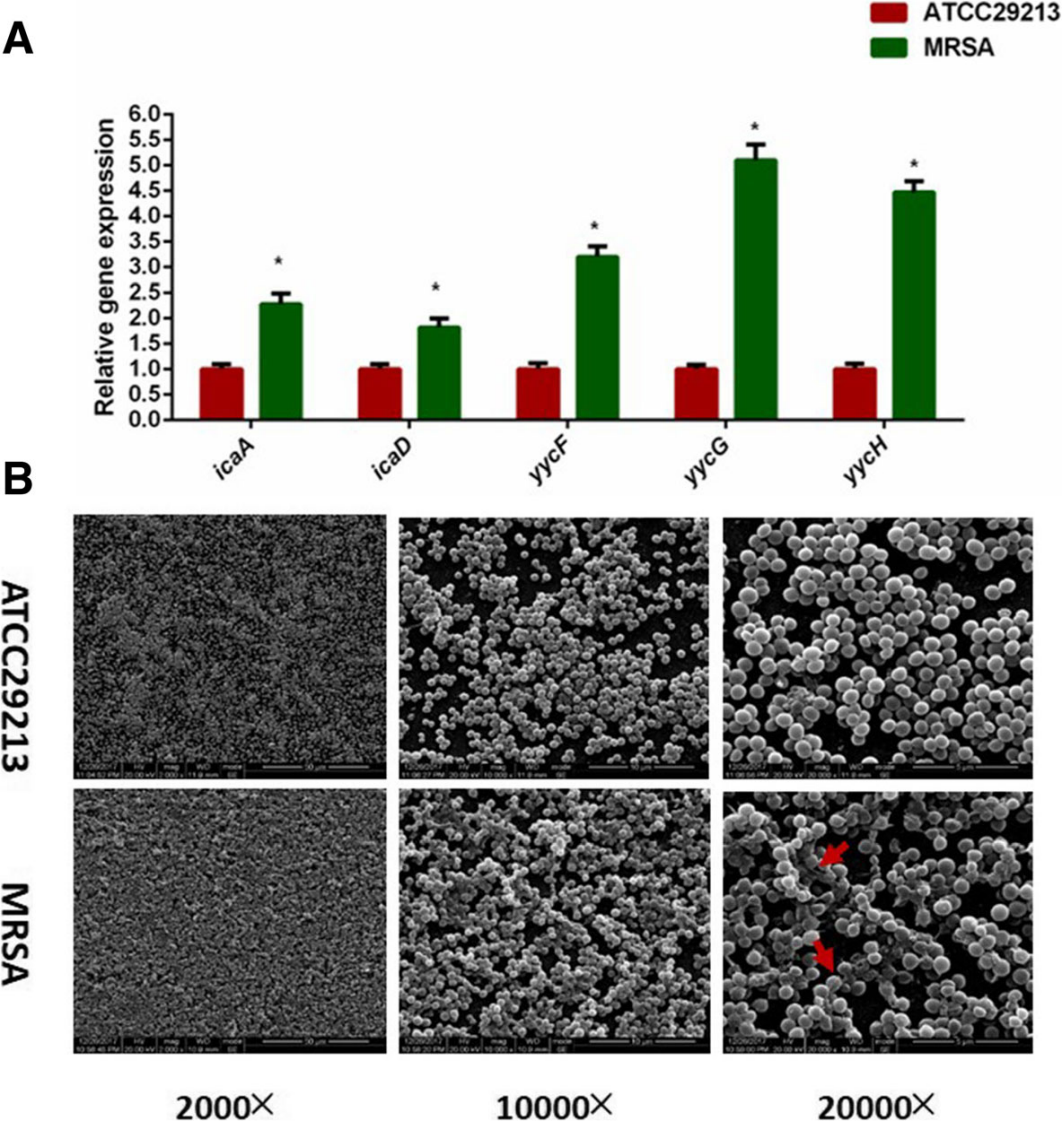
The expression of virulent-associated genes and phenotypic characteristics of *S. aureus.* **a** Quantitative RT-PCR analysis showed the genes transcripts in ATCC29213 and MRSA. Experiments were performed in triplicate and presented as the mean ± standard deviation (*n* = 10; *p < 0.05). **b** Scanning electron microscopy (SEM) observation of the architecture of *S. aureus* 24-h biofilms. Clusters of bacterial cells were surrounded by the EPS matrix in MRSA (red arrows). The ATCC29213 cells seemed to be devoid of EPS matrix in the biofilms

MRSA strains showed elevated expression of virulence-associated genes which affected their growth rates and biofilm formation. Comparative quantitative RT-PCR analysis showed that expression of the *icaA*, *icaD*, *yycF*, *yycG*, and *yycH* transcripts were upregulated in the MRSA strains by 2.2-, 1.8-, 3.2-, 5.1-, and 4.5-folds, respectively, compared to the MSSA strain (Fig. 3a). The morphology of the *S. aureus* biofilms was analyzed using SEM (Fig. 3b). After the introduction of 0.5% glucose, an EPS matrix was seen in the MRSA biofilms surrounding clusters of cells, whereas the biofilms of the MSSA strain showed uneven EPS matrix interspersed with “blank” areas. Taken together, the expression of virulence-associated and biofilm formation genes was altered in the MRSA strains.

### MRSA enhanced biofilm organization and adhesion ability

**Fig. 4:**
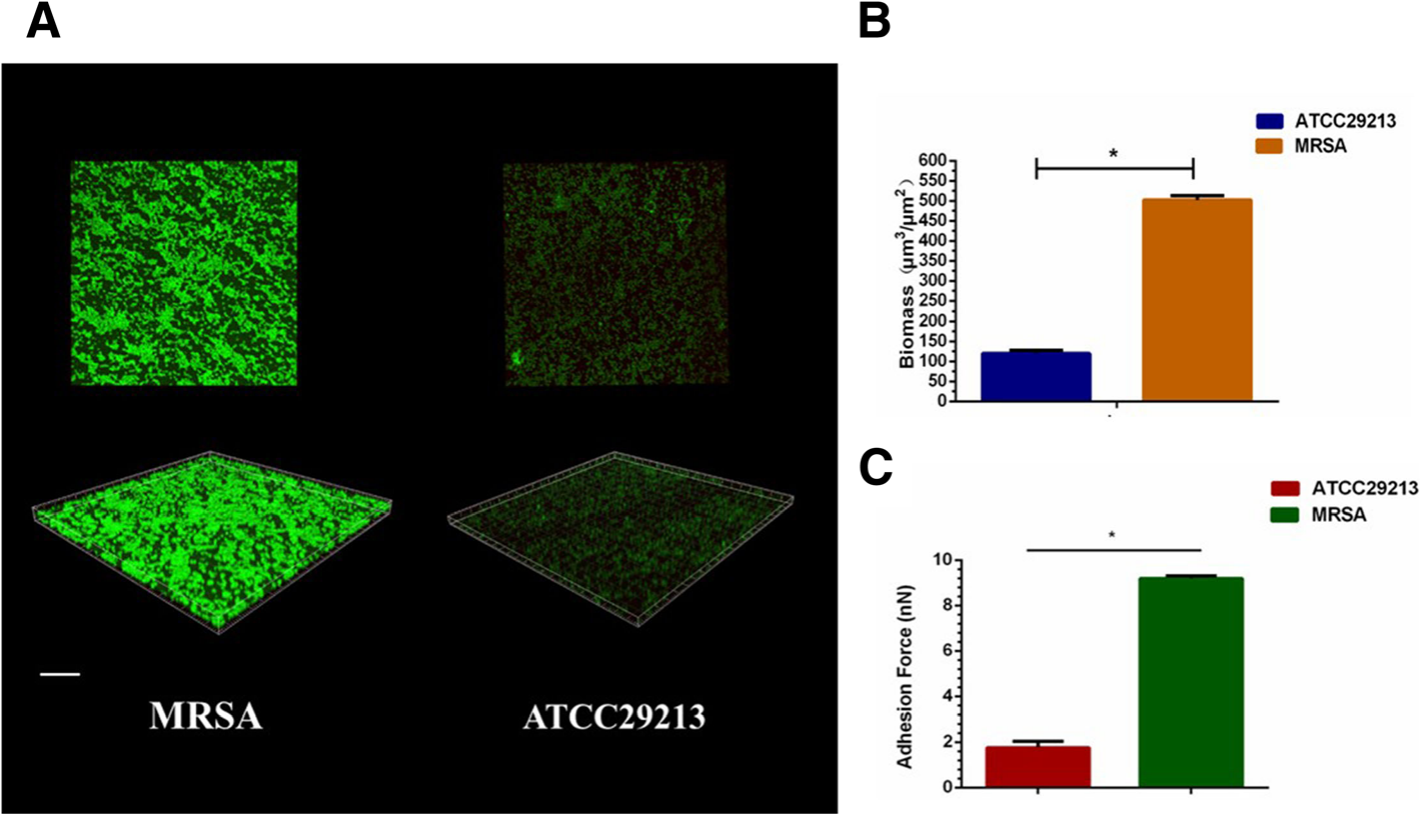
Laser confocal microscopy of EPS matrix in the biofilm architecture. **a** Labeling of the biofilms in the ATCC29213 and MRSA strains in *S. aureus* 24-h biofilms. Green, total bacteria (SYTO 9); scale bars, 100 μm. **b** Quantitative data of bacterial biomass from the biofilms reconstruction (*p < 0.05, *n* = 10). **c** The values of adhesion force of *S. aureus* 24-h biofilms which were obtained from atomic force microscopy (AFM) experiments (*p < 0.05, *n* = 10)

Three-dimensional views obtained by CLSM showed significantly greater bacterial biomass of the MRSA biofilms compared to that of the MSSA strain (Fig. 4a). Furthermore, from the amount of biomass that was obtained, it was highly likely that the biomass volume was inhibited in the MSSA strain (120 ± 8.2 μm^3^/μm^2^; Fig. 4b). With elevated bacterial biomass, the adhesion force between the bacterial cells and the AFM probe was considerable in MRSA biofilms (9.2 ± 0.11 nN; Fig. 4c) while the same was significantly decreased in the MSSA biofilms (1.76 ± 0.28 nN; Fig. 4c).

## Discussion

Sequencing of 16S rRNA amplicons indicated that the infection in chronic osteomyelitis specimens was polymicrobial in nature. The bacterial species detected included five phyla (*Actinobacteria*, *Bacteroidetes*, *Firmicutes*, *Fusobacteria*, *Proteobacteria*). Polymicrobial infections have been observed in previous studies of chronic osteomyelitis of the jaw [18, 28]. In the present study, the phylum *Bacteroidia* made up the major part (82.69%) of the diseased tissues’ microbiota. In terms of microbial diversity, the genus *Porphyromonas* was the most abundant (Fig. 1a). The second most abundant phylum in the chronic osteomyelitis specimens was *Firmicutes* which made up 13% of the microbiota. It has been speculated that the deeper osteomyelitis tissues adapt to the anaerobic environment. This and the polymicrobial nature of the infection make it difficult to identify most of the causative microbes with routine culture-dependent methods [29], underscoring the role of genetic sequencing. Moreover, further analysis of the phylogenetic distribution of bacteria in MSSA osteomyelitis specimens would shed light on the interaction between MRSA and the microbiota in chronic osteomyelitis.

The methicillin-resistant *Staphylococcus aureus* (MRSA) strains on the other hand have been shown to be crucial in such recalcitrant and persistent infections [2]. Therefore, the potential mechanism involved in the pathogenicity of MRSA deserves further investigation. In recent years, an increasing number of studies have investigated the genetic pathways involved in the drug resistance of MRSA strains [30, 31]. However, there is no evidence for any direct interaction between these pathways and the biofilm formation seen in a methicillin-resistant clinical isolate. Among drug-resistant regulatory networks, the two-component signal transduction systems (TCS) are essential for bacterial adaptation, survival, and virulence which contribute to antibiotic resistance [32, 33]. Typically, TCS signal transduction comprises of a membrane-associated histidine kinase and a cytoplasmic response regulator. The histidine kinase recognizes an environmental change, e.g., in pH, osmolarity, or oxidation reduction, and auto-phosphorylates at a conserved histidine residue. Following the transfer of the phosphorylated moiety to the response regulator, the latter can control transcription of target genes. The metabolic processes controlled by TCS include quorum sensing, sporulation, and bacteriocin production in a wide variety of bacteria [34, 35].

In the present study, MRSA isolates from chronic osteomyelitis specimens revealed upregulated levels of *yycF*, *yycG*, and *yycH* genes when compared to the MSSA ATCC29213 strain (Fig. 3a), underscoring their role in MRSA pathogenicity and antibiotic resistance. It was revealed that mutants of YycHI are selected for leading to reduced WalRK activation which was associated with activation and impaired cell wall turnover [30]. In *S. aureus*, WalR is a critical transcriptional regulator which has been shown to influence the expression of genes associated with amino acid biosynthesis, central metabolism, and virulence [36, 37]. In the present study, the MSSA strain showed altered growth pattern with a significantly delayed (4 h) entry into the log phase compared to the MRSA strains (Fig. 2b), suggesting that YycFG affects growth rates.

Polysaccharide intercellular adhesion (PIA), a β-1, 6-linked *N*-acetyl-glucosamine homopolymer that triggers the accumulation of bacterial biofilm, is crucial to the cellular adhesion and pathogenesis of *S. aureus* [23]. PIA is synthesized by enzymes encoded by the *ica* locus [38], and the MRSA strains in this study show an upregulation of *icaA* transcripts (Fig. 3a), indicating an important role of intercellular polysaccharide in *S. aureus* pathogenesis [39]. We found that the biofilm formation decreased by 65% in MSSA compared with MRSA (Fig. 2c, d). This phenotype fits with the easily disrupted biofilm and decreased intercellular polysaccharide matrix accumulation seen in MSSA.

Regarding the association between intercellular polysaccharides layer and biofilm aggregation of *S. aureus* isolates, the AFM is a useful tool to observe bacterial biofilm formation [40]. With accumulated intercellular polysaccharides matrix and enhanced biofilm biomass (Fig. 4a, b), the bacterial adhesion force in MRSA was significantly higher (9.2 ± 0.11 nN) compared to that of MSSA (1.76 ± 0.28 nN, Fig. 4c). Apart from cell adhesion, PIAs are also essential for shaping *S. aureus* biofilm architecture [23]. After the introduction of 0.5% glucose in the culture media, the MRSA cells were covered with reticular intercellular polysaccharides matrix while MSSA biofilms were devoid of these intercellular polysaccharides and had “blank” areas (Fig. 3b). Further studies are needed to validate our findings and elucidate the role of the YycH pathway in MRSA virulence. Within the limitations of this study, the potential mechanisms of the interactions between biofilm organization and expression of YycFG two-component systems should be considered in further investigations. On the other hand, the current study includes the use of single MSSA strain and lacking use of deletion mutants to study the roles of YycFG two-component pathway involved in biofilm formation and bacterial virulence. Therefore, additional information about the in vitro and in vivo studies needs to be explored in the future.

## Conclusion

The 16S rRNA amplicon sequencing confirmed the polymicrobial nature of chronic osteomyelitis, and the MRSA strains from clinical specimens were successfully isolated and identified. Increased expressions of *yycF*, *yycG*, and *yycH* transcripts were observed in MRSA isolates which affected the bacterial growth rates. Furthermore, MRSA biofilms showed an accumulation of intercellular polysaccharides matrix and enhanced biofilm biomass. The MRSA isolate also showed an enhanced expression of the *icaA* gene, suggesting the role of intercellular polysaccharide in *S. aureus* pathogenesis. These findings indicate potential associations between the YycFG pathway and the virulence of clinical MRSA strains and provide a new therapeutic perspective for MRSA-induced chronic osteomyelitis targeting this pathway and disrupting the intercellular polysaccharides matrix.

## Additional file

Additional file 1: Methods and material. DNA extraction, sequencing and bioinformatics analyses. Bacterial strains and growth conditions. Transcription analysis by quantitative RT-PCR. Detection of bacterial growth. Clinical MRSA strains were identified by 16S rRNA sequencing. **Table S1.** Sequences of primers used for qRT-PCR analysis. **Table S2.** Demographical and clinical data of patients with MRSA infections. **Figure S1.** Initial comparison of ATCC29213 and MRSA strains on morphology of *S. aureus*. (A) *S. aureus* biomass was quantified by crystal violet staining and the MRSA strains form more robust biofilm. (B) For crystal violet microtiter assay for determining biofilm biomass, the optical density at 550 nm was read. Data represent ten biological replicates and are presented as the mean ± standard deviation. **Figure S2.** The expression of virulent-associated genes and phenotypic characteristics of *S. aureus*. (PDF 1442 kb)

## Abbreviations

AFM: Atomic force microscopy
BP: Baird-Parker
CLSM: Confocal laser scanning microscope
CV: Crystal violet
EPS: Extracellular polymeric substances
MRSA: Methicillin-resistant *Staphylococcus aureus*
MSSA: Methicillin-sensitive *Staphylococcus aureus*
OTUs: Operational taxonomic units
PIA: Polysaccharide intercellular adhesion
*S. aureus*: *Staphylococcus aureus*
SEM: Scanning electron microscopy
TCS: Two-component regulatory system
