# Supplementary material for: Staphylococcus aureus biofilm organization modulated by YycFG two-component regulatory pathway
Source: J Orthop Surg Res. 2019 Jan 8;14:10. doi: 10.1186/s13018-018-1055-z (PMC6325680; doi:10.1186/s13018-018-1055-z)
Supplement: Supplementary file 1 — Methods and material. DNA extraction, sequencing and bioinformatics analyses. Bacterial strains and growth conditions. Transcription analysis by quantitative RT-PCR. Detection of bacterial growth. Clinical MRSA strains were identified by 16S rRNA sequencing. Table S1. Sequences of primers used for qRT-PCR analysis. Table S2. Demographical and clinical data of patients with MRSA infections. Figure S1. Initial comparison of ATCC29213 and MRSA strains on morphology of S. aureus. (A) S. aureus biomass was quantified by crystal violet staining and the MRSA strains form more robust biofilm. (B) For crystal violet microtiter assay for determining biofilm biomass, the optical density at 550 nm was read. Data represent ten biological replicates and are presented as the mean ± standard deviation. Figure S2. The expression of virulent-associated genes and phenotypic characteristics of S. aureus. (PDF 1442 kb) [file 13018_2018_1055_MOESM1_ESM.pdf]

Appendix and Supplementary materials

Methods and material

DNA extraction, sequencing and bioinformatics analyses

Tissue specimens were centrifuged and the pellets were treated with 1% SDS and 50μg/mL proteinase K (Boehringer, Mannheim, Germany) at 48°C overnight. Total DNA was extracted with phenol/chloroform and precipitated in 100% ethanol. The pelleted DNA was washed twice in 70% ethanol, dissolved in 10mM TRIS buffer (pH 8), and stored at −20°C. An amplicon library from clinical specimens was created by PCR amplification with unique barcoded primers specific to the 16S rRNA V3-V4 gene region; the 338F and 806R primer pair, which contained the 30-mer 5′ -end adapter sequence required for Illumina Hiseq System was used.

Bacterial strains and growth conditions

S. aureus strains were cultured as described previously (23).on tryptic soy agar (TSA) or in broth (TSB) (Oxoid, Basingstoke, United Kingdom) containing 0.5% glucose. Methicillin sodium (50 μg/mL) was added to the media when necessary. The strains were cultured to mid-exponential phase (optical density at 600 nm [OD_{600}] = 0.3) in TSB medium for RNA extraction. To propagate the ATCC29213 and MRSA strains, 50μl of mid-log-phase cells were inoculated in triplicates into 1mL TSB broth containing 0.5% glucose.

Isolation of RNA and cDNA reverse transcription for RT-PCR assays

Briefly, the different strains were cultured overnight in TSB medium containing 0.5% glucose and supplemented with 50 µg/mL methicillin sodium when necessary, diluted with fresh media and again cultured to mid-exponential phase ([OD_{600}] = 0.3). Total RNA was extracted using a MasterPureTM RNA purification Kit (Epicentre Technologies, Epicentre, Madison, WI, USA) in accordance with the recommendations of the manufacturer. Contaminating genomic DNA was removed by digestion with Turbo RNase-free DNase I (Ambion) according to the manufacturer's instructions.

Transcription analysis by quantitative RT-PCR

Real-time PCR was carried out with Quantitect SYBR-Green PCR kit (QIAGEN, Valencia, CA) and Roche Applied LightCycler480 (Roche, Rotkreuz, Switzerland). For each RT- PCR reaction, 20µL of master mixture containing 10µL of SYBR Premix Ex TaqII, 0.5µL of 20µM PCR Forward Primer, 0.5µL of 20µM PCR Reverse Primer, 2.0µL of template cDNA, and 6.0µL of deionized water was placed in each well. The conditions for RT-PCR were as follows: initial denaturation at 95°C for 3 min, followed by 40 cycles each of 5s denaturation at 95°C, 30s primer annealing at 60°C, and dissociation. All reactions were carried out in triplicates. Threshold cycle values (CT) were quantified and the expression of each gene was normalized relative to that of 16sR gene used as an internal reference. Data were calculated according to the $2^{-\Delta\Delta CT}$ method. The differences

between the expression levels of various genes were considered significant at a cut-off of 2-fold change to baseline.

Detection of bacterial growth

The bacterial strains were cultured at 37℃ in TSB medium and growth curves were plotted by measuring OD_{600} at one hour intervals. Each sample was technically replicated in three times. The data represents for the average values of OD_{600} absorbance. *S. aureus* biofilm growth was established as follows: glass cover slips were placed inside 12-well plates, and each well was inoculated with 10μL exponential phase culture (OD_{600} = 0.3) in 1 mL TSB broth containing 0.5% glucose. After anaerobic incubation for 24h, the planktonic suspensions were removed and the biofilms growing on the cover slips were washed three times with phosphate-buffered saline (PBS, pH 7.2) to remove non-adherent cells. The biofilms were washed twice with PBS buffer and fixed with 2.5% glutaraldehyde for 4 hours. They were then serially dehydrated with increasingly concentrated ethanol solutions (30%, 50%, 70%, 80%, 95%, and 100%), dried to critical-point with liquid CO2, and coated with gold powder. Scanning electron micrographs of the biofilms were then obtained with an SEM (Inspect Hillsboro, OR, USA).

Clinical MRSA strains were identified by 16S rRNA sequencing

1497bp

CTGGCTCAGGATGAACGCTGGCGGCGTGCCTAATACATGCAAGTCGAGCGAACGGACGAGAAGCTTGCTTCTCTGATGTTAGCGGCGGACGGGTGAGTAACACGTGGATAACCTACCTATAAGACTGGGATAACTTCGGGAAACCGGAGCTAATACCGGATAATATTTTGAACCGCATGGTTCAAAAGTGAAAGACGGTCTTGCTGTCACTTATAGATGGATCCGCGCTGCATTAGCTAGTTGGTAAGGTAACGGCTTACCAAGGCAACGATGCATAGCCGACCTGAGAGGGTGATCGGCCACACTGGAACTGAGACACGGTCCAGACTCCTACGGGAGGCAGCAGTAGGGAATCTTCCGCAATGGGCGAAAGCCTGACGGAGCAACGCCGCGTGAGTGATGAAGGTCTTCGGATCGTAAAACTCTGTTATTAGGGAAGAACATATGTGTAAGTAACTGTGCACATCTTGACGGTACCTAATCAGAAAGCCACGGCTAACTACGTGCCAGCAGCCGCGGTAATACGTAGGTGGCAAGCGTTATCCGGAATTATTGGGCGTAAAGCGCGCGTAGGCGGTTTTTTAAGTCTGATGTGAAAGCCCACGGCTCAACCGTGGAGGGTCATTGGAAACTGGAAAACTTGAGTGCAGAAGAGGAAAGTGGAATTCCATGTGTAGCGGTGAAATGCGCAGAGATATGGAGGAACACCAGTGGCGAAGGCGACTTTCTGGTCTGTAACTGACGCTGATGTGCGAAAGCGTGGGGATCAAACAGGATTAGATACCCTGGTAGTCCACGCCGTAAACGATGAGTGCTAAGTGTTAGGGGGTTTCCGCCCCTTAGTGCTGCAGCTAACGCATTAAGCACTCCGCCTGGGGAGTACGACCGCAAGGTTGAAACTCAAAGGAATTGACGGGGACCCGCACAAGCGGTGGAGCATGTGGTTTAATTCGAAGCAACGCGAAGAACCTTACCAAATCTTGACATCCTTTGACAACTCTAGAGATAGAGCTTTCCCCTTCGGGGGACAAAGTGACAGGTGGTGCATGGTTGTCGTCAGCTCGTGTCGTGAGATGTTGGGTTAAGTCCCGCAACGAGCGCAACCCTTAAGCTTAGTTGCCATCATTAAGTTGGGCACTCTAAGTTGACTGCCGGTGACAAACCGGAGGAAGGTGGGGATGACGTCAAATCATCATGCCCCTTATGATTTGGGCTACACACGTGCTACAATGGACAATACAAAGGGCAGCGAAACCGCGAGGTCAAGCAAATCCCATAAAGTTGTTCTCAGTTCGGATTGTAGTCTGCAACTCGACTACATGAAGCTGGAATCGCTAGTAATCGTAGATCAGCATGCTACGGTGAATACGTTCCCGGGTCTTGTACACACCGCCCGTCACACCACGAGAGTTTGTAACACCCGAAGCCGGTGGAGTAACCTTTTAGGAGCTAGCCGTCGAAGGTGGGACAAATGATTGGGGTGAAGTCGTAACAAGG

Description	Max score	Total score	Query cover	E value	Ident	Accession
Staphylococcus aureus strain FORC_045, complete genome	2765	13794	100%	0.0	100%	CP017115.1
Staphylococcus aureus strain ISU935, complete genome	2765	16508	100%	0.0	100%	CP017090.1
Staphylococcus aureus DNA, almost complete genome, strain: No.10	2765	16554	100%	0.0	100%	AP015012.1
Staphylococcus aureus strain FORC_039, complete genome	2765	16565	100%	0.0	100%	CP015817.1
Staphylococcus aureus strain BA01611, complete genome	2765	13776	100%	0.0	100%	CP019945.1
Staphylococcus aureus subsp. aureus strain ATCC 6538, complete genome	2765	16560	100%	0.0	100%	CP020020.1

Description	Max score	Total score	Query cover	E value	Ident	Accession
Staphylococcus aureus strain SR434, complete genome	2765	16543	100%	0.0	100%	CP019563.1
Staphylococcus aureus strain FORC_026, complete genome	2765	16554	100%	0.0	100%	CP013132.1
Staphylococcus aureus subsp. aureus strain 2148.N, complete genome	2765	16534	100%	0.0	100%	CP016856.1
Staphylococcus aureus strain 08-02119, complete genome	2765	16560	100%	0.0	100%	CP015645.1
Staphylococcus aureus strain NCCP14562, complete genome	2765	13811	100%	0.0	100%	CP013955.1

Project: S1678-1.sqd Contig 1

10 20 30 40 50 60 70

CTGGCTCAGGATGAACGCTGGCGGCGTGCCTAATACATGCAAGTCGAGCGAACGGACGAGAAGCTTGCTTCTCTGATG

8.21xS1678-1-518R_C03.ab1(1>500) ←

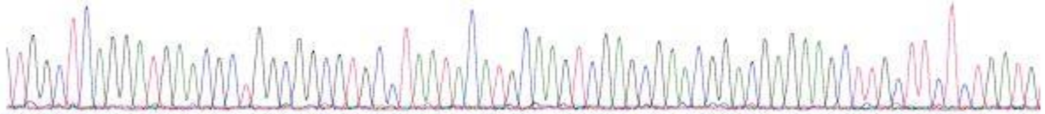

CTGGCTCAGGATGAACGCTGGCGGCGTGCCTAATACATGCAAGTCGAGCGAACGGACGAGAAGCTTGCTTCTCTGATG

80 90 100 110 120 130 140 150

TTAGCGGCGGACGGGTGAGTAACACGTGGATAACCTACCTATAAGACTGGGATAACTTCGGGAAACCGGAGCTAATAC

8.21xS1678-1-518R_C03.ab1(1>500) ←

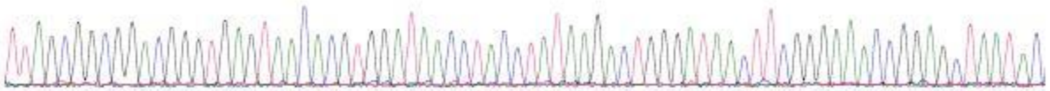

TTAGCGGCGGACGGGTGAGTAACACGTGGATAACCTACCTATAAGACTGGGATAACTTCGGGAAACCGGAGCTAATAC

160 170 180 190 200 210 220 230

CGGATAATATTTTGAACCGCATGGTTCAAAAGTGAAAGACGGTCTTGCTGTCACTTATAGATGGATCCGCGCTGCATT

8.21xS1678-1-518R_C03.ab1(1>500) ←

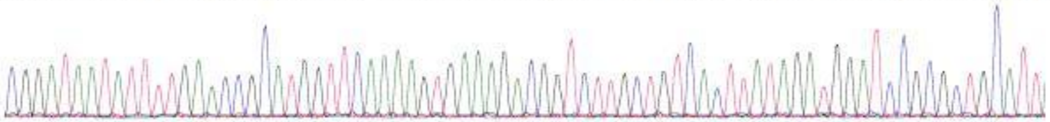

CGGATAATATTTTGAACCGCATGGTTCAAAAGTGAAAGACGGTCTTGCTGTCACTTATAGATGGATCCGCGCTGCATT

240 250 260 270 280 290 300 310

AGCTAGTTGGTAAGGTAACGGCTTACCAAGGCAACGATGCATAGCCGACCTGAGAGGGTGATCGGCCACACTGGAACT

8.21xS1678-1-518R_C03.ab1(1>500) ←

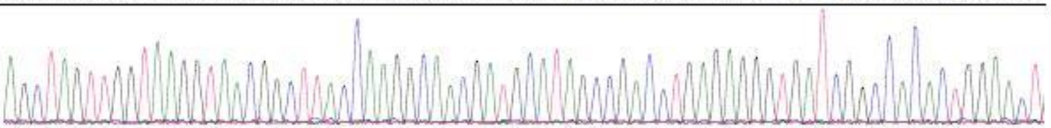

AGCTAGTTGGTAAGGTAACGGCTTACCAAGGCAACGATGCATAGCCGACCTGAGAGGGTGATCGGCCACACTGGAACT

57

2017[illegible]8[illegible]22[illegible] 9:28 Page 2
Project: S1678-1.sqd Contig 1

320 330 340 350 360 370 380 390

GAGACACGGTCCAGACTCCTACGGGAGGCAGCAGTAGGGAATCTTCCGCAATGGGCGAAAGCCTGACGGAGCAACGCC

8.21xS1678-1-518R_C03.ab1(1>500) ←

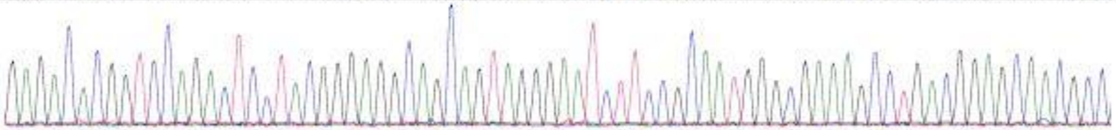

GAGACACGGTCCAGACTCCTACGGGAGGCAGCAGTAGGGAATCTTCCGCAATGGGCGAAAGCCTGACGGAGCAACGCC

400 410 420 430 440 450 460

GCGTGAGTGATGAAGGTCTTCGGATCGTAAAACTCTGTTATTAGGGAAGAACATATGTGTAAGTAACTGTGCACATCT

8.21xS1678-1-518R_C03.ab1(1>500) ←

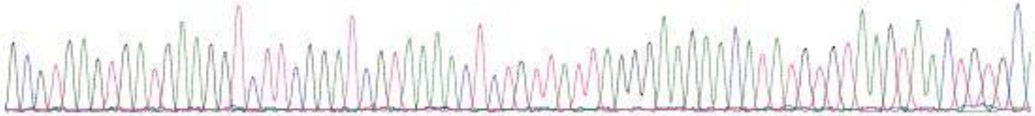

GCGTGAGTGATGAAGGTCTTCGGATCGTAAAACTCTGTTATTAGGGAAGAACATATGTGTAAGTAACTGTGC

8.21xS1678-1-1100R_G03.ab1(1>753) ←

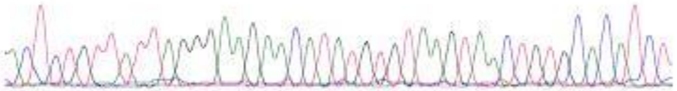

ACTCTGTTATTAGGGAAGAACATATGTGTAAGTAACTGTGCACATCT

470 480 490 500 510 520 530 540

TGACGGTACCTAATCAGAAAGCCACGGCTAACTACGTGCCAGCAGCCGCGGTAATACGTAGGTGGCAAGCGTTATCCG

8.21xS1678-1-1100R_G03.ab1(1>753) ←

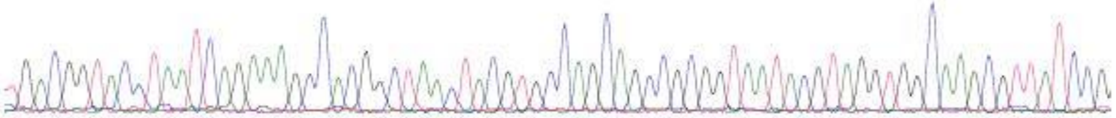

TGACGGTACCTAATCAGAAAGCCACGGCTAACTACGTGCCAGCAGCCGCGGTAATACGTAGGTGGCAAGCGTTATCCG

550 560 570 580 590 600 610 620

GAATTATTGGGCGTAAAGCGCGCGTAGGCGGTTTTTTAAGTCTGATGTGAAAGCCCACGGCTCAACCGTGGAGGGTCA

8.21xS1678-1-1100R_G03.ab1(1>753) ←

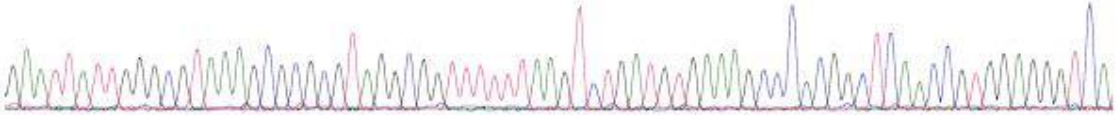

GAATTATTGGGCGTAAAGCGCGCGTAGGCGGTTTTTTAAGTCTGATGTGAAAGCCCACGGCTCAACCGTGGAGGGTCA

630 640 650 660 670 680 690 700

TTGGAAACTGGAAAACTTGAGTGCAGAAGAGGAAAGTGGAATTCCATGTGTAGCGGTGAAATGCGCAGAGATATGGAG

8.21xS1678-1-1100R_G03.ab1(1>753) ←

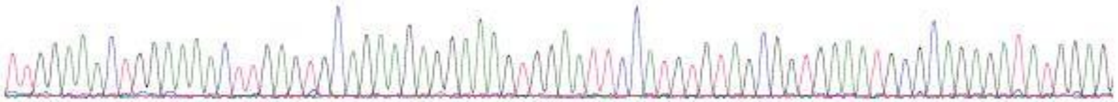

TTGGAAACTGGAAAACTTGAGTGCAGAAGAGGAAAGTGGAATTCCATGTGTAGCGGTGAAATGCGCAGAGATATGGAG

710 720 730 740 750 760 770 780

GAACACCAGTGGCGAAGGCGACTTTCTGGTCTGTAACTGACGCTGATGTGCGAAAGCGTGGGGATCAAACAGGATTAG

8.21xS1678-1-1100R_G03.ab1(1>753) ←

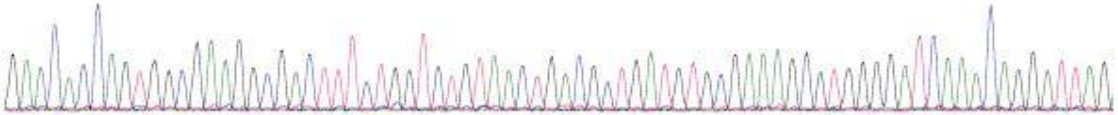

GAACACCAGTGGCGAAGGCGACTTTCTGGTCTGTAACTGACGCTGATGTGCGAAAGCGTGGGGATCAAACAGGATTAG

790 800 810 820 830 840 850

ATACCCTGGTAGTCCACGCCGTAAACGATGAGTGCTAAGTGTTAGGGGGTTTCCGCCCCTTAGTGCTGCAGCTAACGC

8.21xS1678-1-1100R_G03.ab1(1>753) ←

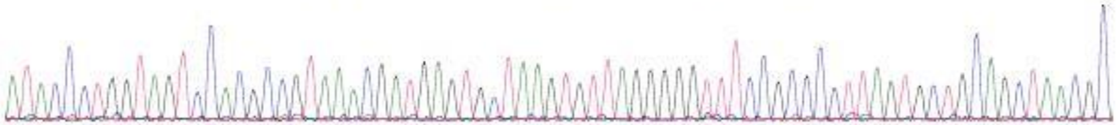

ATACCCTGGTAGTCCACGCCGTAAACGATGAGTGCTAAGTGTTAGGGGGTTTCCGCCCCTTAGTGCTGCAGCTAACGC

860 870 880 890 900 910 920 930

ATTAAGCACTCCGCCTGGGGAGTACGACCGCAAGGTTGAAACTCAAAGGAATTGACGGGGACCCGCACAAGCGGTGGA

8.21xS1678-1-1100R_G03.abl(1>753) ←

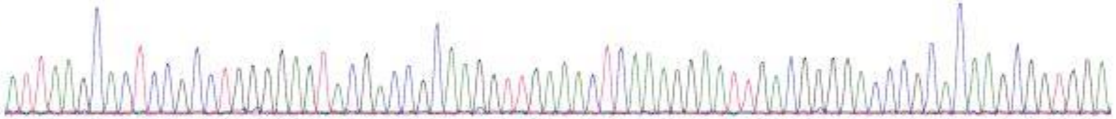

ATTAAGCACTCCGCCTGGGGAGTACGACCGCAAGGTTGAAACTCAAAGGAATTGACGGGGACCCGCACAAGCGGTGGA

940 950 960 970 980 990 1000 1010

GCATGTGGTTTAATTCGAAGCAACGCGAAGAACCTTACCAAATCTTGACATCCTTTGACAACTCTAGAGATAGAGCTT

8.21xS1678-1-1100R_G03.abl(1>753) ←

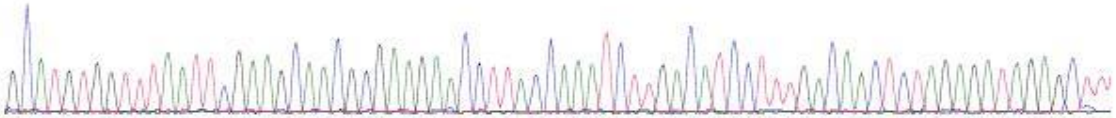

GCATGTGGTTTAATTCGAAGCAACGCGAAGAACCTTACCAAATCTTGACATCCTTTGACAACTCTAGAGATAGAGCTT

8.21xS1678-1-926F_C04.abl(1>530) →

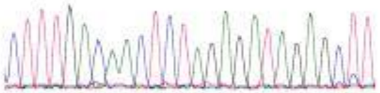

CTTTGACAACTCTAGAGATAGAGCTT

1020 1030 1040 1050 1060 1070 1080 1090

TCCCCTTCGGGGGACAAAGTGACAGGTGGTGCATGGTTGTCGTCAGCTCGTGTCGTGAGATGTTGGGTTAAGTCCCGC

8.21xS1678-1-1100R_G03.abl(1>753) ←

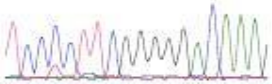

TCCCCTTCGGGGGACAAA

8.21xS1678-1-926F_C04.abl(1>530) →

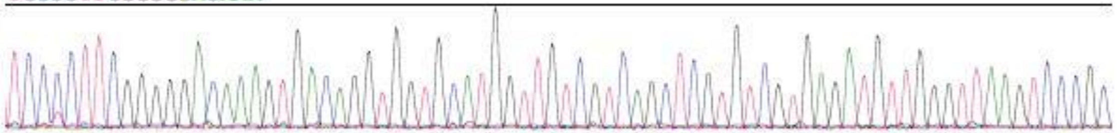

TCCCCTTCGGGGGACAAAGTGACAGGTGGTGCATGGTTGTCGTCAGCTCGTGTCGTGAGATGTTGGGTTAAGTCCCGC

1100 1110 1120 1130 1140 1150 1160 1170

AACGAGCGCAACCCTTAAGCTTAGTTGCCATCATTAAGTTGGGCACTCTAAGTTGACTGCCGGTGACAAACCGGAGGA

8.21xS1678-1-926F_C04.ab1(1>530) →

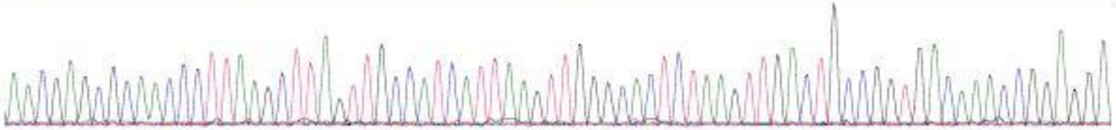

AACGAGCGCAACCCTTAAGCTTAGTTGCCATCATTAAGTTGGGCACTCTAAGTTGACTGCCGGTGACAAACCGGAGGA

1180 1190 1200 1210 1220 1230 1240

AGGTGGGGATGACGTCAAATCATCATGCCCCTTATGATTTGGGCTACACACGTGCTACAATGGACAATACAAAGGGCA

8.21xS1678-1-926F_C04.ab1(1>530) →

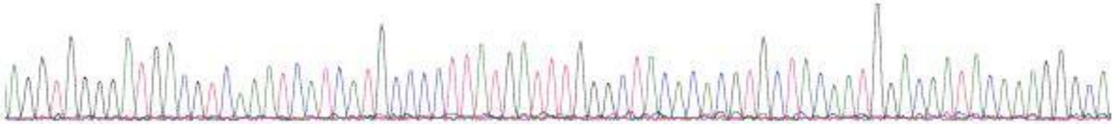

AGGTGGGGATGACGTCAAATCATCATGCCCCTTATGATTTGGGCTACACACGTGCTACAATGGACAATACAAAGGGCA

1250 1260 1270 1280 1290 1300 1310 1320

GCGAAACCGCGAGGTCAAGCAAATCCCATAAAGTTGTTCTCAGTTCGGATTGTAGTCTGCAACTCGACTACATGAAGC

8.21xS1678-1-926F_C04.ab1(1>530) →

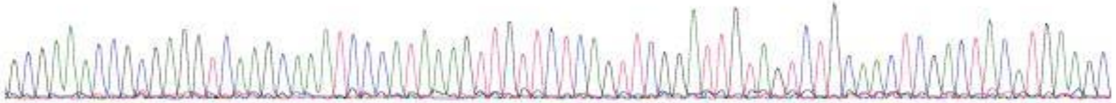

GCGAAACCGCGAGGTCAAGCAAATCCCATAAAGTTGTTCTCAGTTCGGATTGTAGTCTGCAACTCGACTACATGAAGC

1330 1340 1350 1360 1370 1380 1390 1400

TGGAATCGCTAGTAATCGTAGATCAGCATGCTACGGTGAATACGTTCCCGGGTCTTGTACACACCGCCCGTCACACCA

8.21xS1678-1-926F_C04.ab1(1>530) →

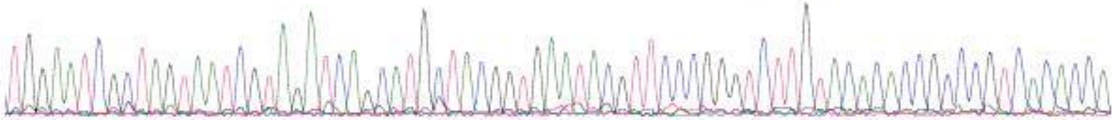

TGGAATCGCTAGTAATCGTAGATCAGCATGCTACGGTGAATACGTTCCCGGGTCTTGTACACACCGCCCGTCACACCA

1410 1420 1430 1440 1450 1460 1470 1480

CGAGAGTTTGTAACACCCGAAGCCGGTGGAGTAACCTTTTAGGAGCTAGCCGTCGAAGGTGGGACAAATGATTGGGGT

8.21xS1678-1-926F_C04.abl(1>530) →

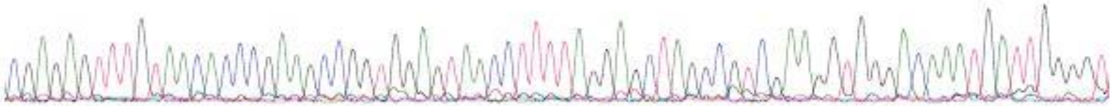

CGAGAGTTTGTAACACCCGAAGCCGGTGGAGTAACCTTTTAGGAGCTAGCCGTCGAAGGTGGGACAAATGATTGGGGT

1490

GAAGTCGTAACAAGG

8.21xS1678-1-926F_C04.abl(1>530) →

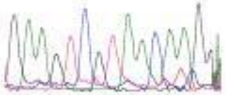

GAAGTCGTAACAAGG

Table 1. Sequences of primers used for qRT-PCR analysis

Primers	sequence 5’-3’ (Forward/Reverse)	Reference
**RT-qPCR**		
icaA	5’- GATTATGTAATGTGCTTGGA -3’/<br>5’- ACTACTGCTGCGTTAATAAT - 3’	This study
icaD	5’- ATGGTCAAGCCCAGACAGAG -3’/<br>5’- CGTGTTTTCAACATTTAATGCAA -3’	This study
yycF	5’ - TGGCGAAAGAAGACATCA -3’/<br>5’ – AACCCGTTACAAATCCTG- 3’	This study
yycG	5’ - CGGGGCGTTCAAAAGACTTT -3’/<br>5’ - TCTGAACCTTTGAACACACGT -3’	This study
yycH	5’ - TCAGTCAGGCGAGCTAACAT -3’/<br>5’ –CGCTAAGCTTGAACGTACAGA -3’	This study
16sr	5’ - GTAGGTGGCAAGCGTTATCC -3’/<br>5’ –CGCACATCAGCGTCAACA-3’	This study

Table2. Demographical and clinical data of patients with MRSA infections.

Patient	Date of MRSA isolation	Age	Sex	Etiology	Infection site
1	May 2016	44	Male	Trauma	Left tibial
2	May 2016	61	Male	Diabetic foot	Right calcaneus
3	May 2016	32	Female	Trauma	Right tibial
4	May 2016	55	Female	Trauma	Right calcaneus
5	July 2016	28	Female	Diabetic foot	Left calcaneus
6	July 2016	45	Female	Trauma	Right femur
7	July 2016	39	Male	Trauma	Left tibial
8	July 2016	62	Male	Trauma	Left femur
9	July 2016	28	Male	Trauma	Right calcaneus
10	September 2016	33	Male	Trauma	Right tibial
11	September 2016	54	Male	Trauma	Left tibial
12	October 2016	23	Female	Trauma	Left femur
13	October 2016	35	Male	Trauma	Left calcaneus

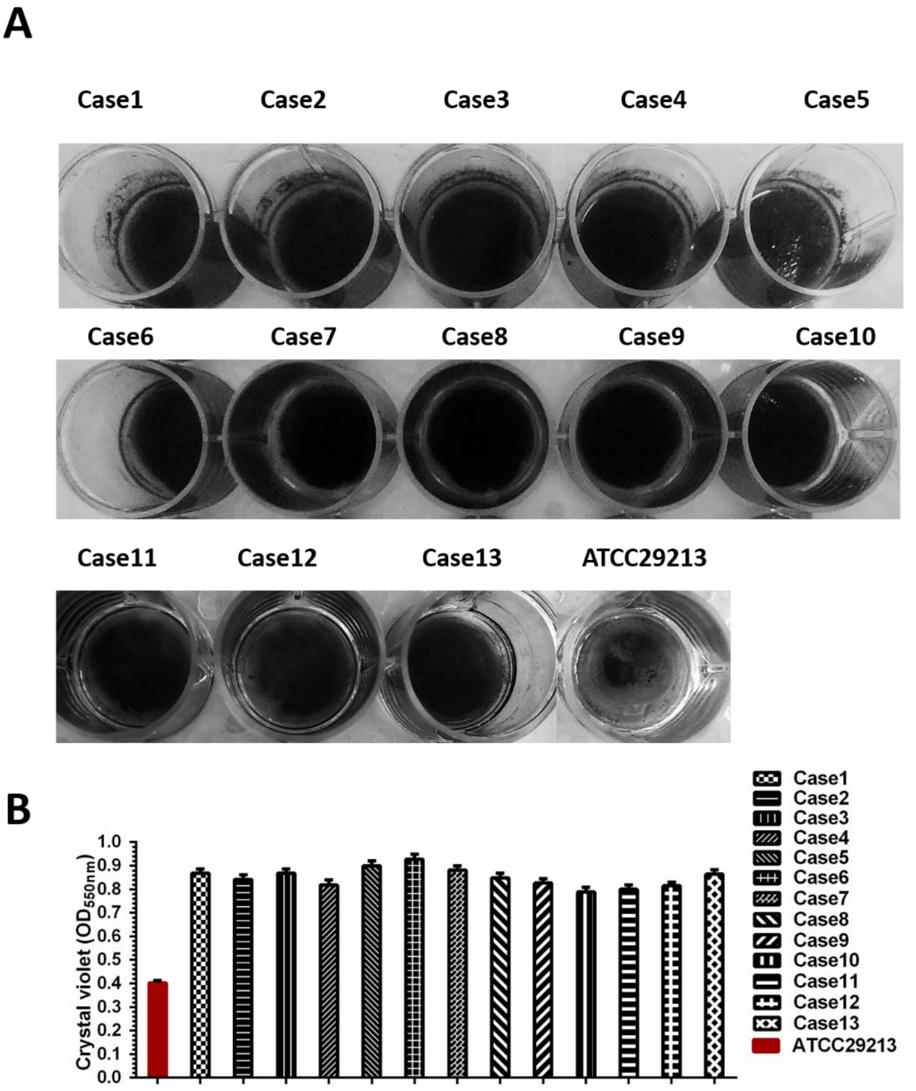

SFig.1 Initial comparison of ATCC29213 and MRSA strains on morphology of *S. aureus*

(A) *S. aureus* biomass was quantified by crystal violet staining and the MRSA strains form more robust biofilm. **(B)** For crystal violet microtiter assay for determining biofilm biomass, the optical density at 550 nm was read. Data represent ten biological replicates and are

presented as the mean ± standard deviation.

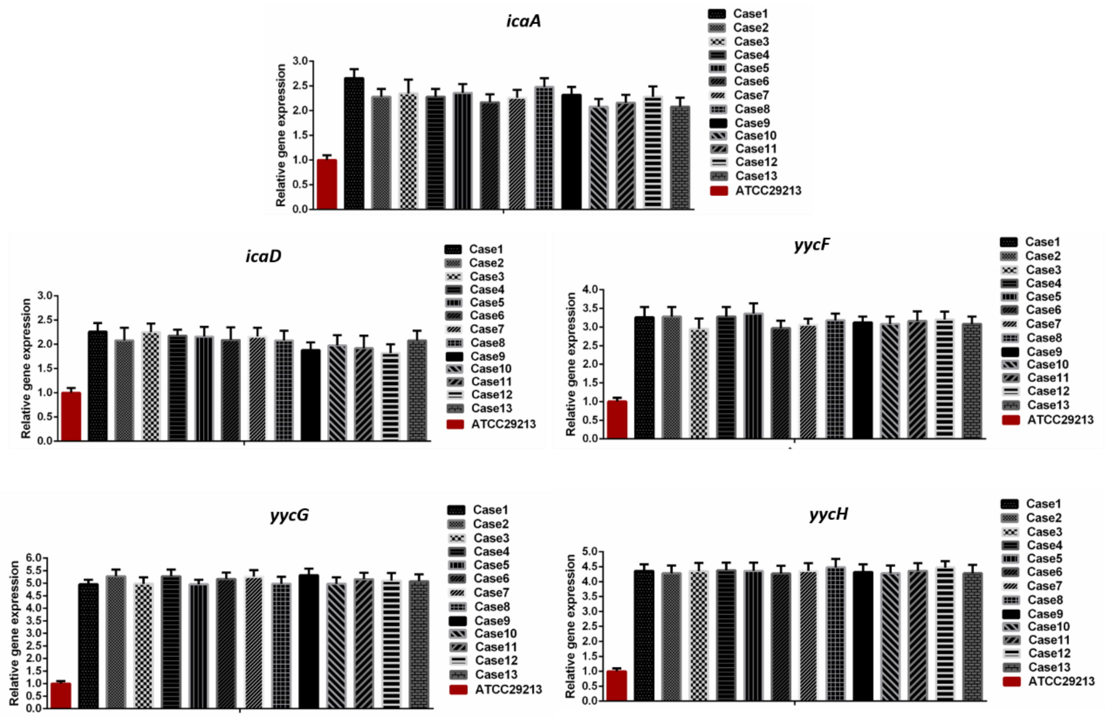

SFig.2 The expression of virulent-associated genes and phenotypic characteristics of *S. aureus*.
